# The government of Kenya cash transfer for orphaned and vulnerable children: cross-sectional comparison of household and individual characteristics of those with and without

**DOI:** 10.1186/1472-698X-14-25

**Published:** 2014-09-20

**Authors:** David Ayuku, Lonnie Embleton, Julius Koech, Lukoye Atwoli, Liangyuan Hu, Samuel Ayaya, Joseph Hogan, Winstone Nyandiko, Rachel Vreeman, Allan Kamanda, Paula Braitstein

**Affiliations:** 1 College of Health Sciences, School of Medicine, Department of Behavioral Sciences, Moi University, Eldoret, Kenya; 2 College of Health Sciences, School of Medicine, Department of Medicine, Moi University, Eldoret, Kenya; 3 Academic Model Providing Access to Healthcare (AMPATH), Eldoret, Kenya; 4 College of Health Sciences, School of Medicine, Department of Mental Health, Moi University, Eldoret, Kenya; 5 Department of Biostatistics, School of Public Health, Brown University, Providence, RI 02912, USA; 6 College of Health Sciences, School of Medicine, Department of Child Health and Pediatrics, Moi University, Eldoret, Kenya; 7 School of Medicine, Department of Pediatrics, Indiana University, Indianapolis, USA; 8 Moi Teaching and Referral Hospital, Eldoret, Kenya; 9 School of Medicine, Department of Medicine, Indiana University, Indianapolis, USA; 10 Dalla Lana School of Public Health, University of Toronto, Toronto, Canada

**Keywords:** Orphans, Africa, Cash transfer, Nutrition, Food security, Mental health

## Abstract

**Background:**

The ‘Cash Transfer to Orphans and Vulnerable Children’ (CT-OVC) in Kenya is a government-supported program intended to provide regular and predictable cash transfers (CT) to poor households taking care of OVC. CT programs can be an effective means of alleviating poverty and facilitating the attainment of an adequate standard of living for people’s health and well-being and other international human rights. The objective of this analysis was to compare the household socioeconomic status, school enrolment, nutritional status, and future outlook of orphaned and separated children receiving the CT compared to those not receiving a CT.

**Methods:**

This project analyzes baseline data from a cohort of orphaned and separated children aged <19 years and non-orphaned children living in 300 randomly selected households (HH) in 8 Locations of Uasin Gishu County, Kenya. Baseline data were analyzed using multivariable logistic and Poisson regression comparing children in CT-HH vs. non-CT HH. Odds ratios are adjusted (AOR) with 95% confidence intervals (CI) for guardian age and sex, child age and sex, and intra-HH correlation.

**Results:**

Included in this analysis were data from 1481 children and adolescents in 300 HH (503 participants in CT, 978 in non-CT households). Overall there were 922 (62.3%) single orphans, 324 (21.9%) double orphans, and 210 (14.2%) participants had both parents alive and were living with them. Participants in CT-HH were less likely to have ≥2 pairs of clothes compared to non-CT HH (AOR: 0.32, 95% CI: 0.16-0.63). Those in CT HH were less likely to have missed any days of school in the preceding month (AOR: 0.62, 95% CI: 0.42-0.94) and those aged <1-18 years in CT-HH were less likely to have height stunting for their age (AOR: 0.65, 95% CI: 0.47-0.89). Participants aged at least 10 years in CT-HH were more likely to have a positive future outlook (AOR: 1.72, 95% CI: 1.12-2.65).

**Conclusions:**

Children and adolescents in households receiving the CT-OVC appear to have better nutritional status, school attendance, and optimism about the future, compared to those in households not receiving the CT, in spite of some evidence of continued material deprivation. Consideration should be given to expanding the program further.

## Background

Kenya’s most vulnerable families caring for orphaned and separated children are unable to uphold many of their basic human rights [1]. These families lack social security and a standard of living adequate for their health and well-being, including food, clothing, housing, medical care, and necessary social services [1] that are basic human rights as outlined in the Universal Declaration of Human Rights [2] and the Convention on the Rights of the Child [3]. Kenya’s population is currently 43 million, 75-80% of whom live in rural areas [4]. With over 50% of the population below 15 years of age, Kenya faces a high dependency burden, which places pressing demands on social services including education and health care [4,5]. Kenya is among the world’s 50 poorest countries, ranking 145 out of 187 countries on the 2013 Human Development Index [6].There are approximately 2.4 million orphaned children in Kenya, of whom roughly 50% have been orphaned by the HIV epidemic [5]. A majority of orphaned children and adolescents in Kenya live in extreme poverty, often with relatives or guardians of limited means [1]. Grandparents, and primarily grandmothers, are often the primary caretaker for orphaned children [1,7,8]. Despite the steady growth of the economy, more than a half of the country’s population live below the poverty line [4]. The most vulnerable are families and children living in the urban and peri-urban slums, in the arid lands of northern Kenya and in areas of the country worst affected by HIV.

Strengthening the capacity of households to care for orphaned and vulnerable children (OVC) within the community is the key strategic response in addressing the OVC crisis. The Cash Transfer to Orphans and Vulnerable Children (CT-OVC) is a government social support program which provides regular and predictable (unconditional) cash transfers to poor households taking care of orphans and vulnerable children. The main objective of the CT-OVC program is to encourage fostering and retention of OVC within their families and communities as well as to enhance their human capital development [9]. The Kenya CT-OVC program started in 2004 and currently supports 151,243 households in 69 districts, translating to support for over 750,000 OVC nationwide [10]. This includes >1800 households in Uasin Gishu (UG) County since the program was rolled out in this region in 2007. Enrolled households receive a cash payment of KSH. 1500/= (approximately $17 USD) per month paid every two months through the Kenya Post Office or Equity Bank [9].

Cash transfer programs can be an effective means of alleviating poverty and facilitating the attainment of an adequate standard of living for people’s health and well-being and other international human rights [11-13]. An independent report for the UNOHCR found that cash transfer programs can assist governments in complying with children’s human rights outlined in the Convention on the Rights of the Child and that these programs can be an effective tool in reducing child poverty. However, the report notes that the evaluation of cash-transfer programs often lack a child-focus [11]. A World Bank report on conditional cash transfers found that these programs had significant impacts on poverty, educational enrollment and moderate improvement of uptake of preventative health services [14]. The evidence regarding the impact of conditional compared to unconditional cash transfers and financial incentives for child health interventions remains unclear [15]. However, programs with conditionalities on participation demonstrated a moderate improvement in health outcomes [15]. In Kenya, beneficiaries of the unconditional CT-OVC program may have improved nutritional status [16] and reduced sexual debut [17]; however there is a lack of literature regarding the impact of Kenya’s CT-OVC program on household and child health outcomes. This study addresses this gap by evaluating associations between household socio-economic and individual health indicators of participants enrolled in the CT-OVC program versus those who are not. As the aim of the CT-OVC is to support househods caring for orphaned children, this study sought to examine whether households receiving the CT-OVC and their orphaned and non-orphaned residents 18 years and below in UG County, Kenya were comparable to households not receiving it in terms of cross-sectional household socio-economic and individual health indicators. We specifically compared household socioeconomic characteristics, the school attendance, nutritional status, and the future outlook of children and adolescents living within them.

## Methods

### Study setting

UG County is one of the 47 counties of Kenya, with its headquarters in Eldoret, about 350 kilometers northwest of Kenya’s capital city, Nairobi. In 2010, UG County had approximately 894,179 individuals from 202,291 households, of whom 41.5% were aged 14 years or less [18]. The majority of the UG County population (61.4%) reside in rural settings [19], comparable to the rest of Kenya (67.7%), but somewhat less than the East African average (77.3%) [20]. Approximately 51.3% of the population in UG County live below the Kenyan poverty line [19]. The city of Eldoret is the County’s capital, administrative and commercial center. Eldoret has a total population of 289,389 and is currently, the 5th largest city in the country.

### OSCAR’s health and well-being project

The Orphaned and Separated Children’s Assessments Related to their (OSCAR’s) Health and Well-Being Project is a 5-year longitudinal cohort study evaluating the effects of different care environments on the physical and mental health outcomes of orphaned and separated children aged 18 years of age or less. The study intends to describe these care environments, determine whether they are able to meet the basic socioeconomic needs of the resident children, and examine the effect of care environment on resident children’s physical and mental health over time. The study began enrolling participants in June 2010.

### Human subjects protection

This study was approved by the Moi University College of Health Sciences and Moi Teaching and Referral Hospital Institutional Research and Ethics Committee and the Indiana University Institutional Review Board. Written informed consent was provided by the head of household, Director of Charitable Children’s Institutions (CCI’s), or in the case of the street youth, by the District Children’s Officer (DCO). Individual written informed assent was provided by each child aged 7 years and above. Fingerprints were used for both children and guardians who were unable to sign or write their name. Verbal assent was provided by children under 7 years but old enough to have a basic understanding of the study purpose and study procedures. The consent and assent processes were documented through the use of consent and assent notes.

### Study population

The project follows a cohort of orphaned and separated children from communities within 8 administrative Locations, and includes 300 households, 19 Charitable Children’s Institutions (CCI’s), and 100 street-involved children and youth in UG County of western Kenya [21]. The present analysis was restricted to baseline data collected from June 2010-April 2013 from the 300 households and individual participants living in these households.

### Eligibility, sampling and recruitment

The project aimed to randomly sample 300 households within eight locations representing families caring for orphaned and separated children in the UG County. In order to obtain a representative sample of households caring for orphans in UG County, the project utilized three sampling arms: cash-transfer (CT) households, non-cash transfer households from the same sub-Location (SSL), and non-cash transfer households from a different sub-Location (DSL). The CT program targets sub-locations that are the most socioeconomically deprived. The CT-OVC program was rolled out in UG County in 2007. Sub-Locations are administrative boundaries within Locations and are headed by an Assistant Chief. 100 households were sampled from each category (CT, SSL, and DSL) and weighted to reflect the number of households required per location based on the number of households in each Location caring for orphaned children as provided by the local officials including the DCO, to ensure appropriate distribution.

The DCO oversees the government CT program and provided the study lists of households receiving the government subsidy in each location. In the planning phases of the project, the DCO was explicit that he wished to conduct an evaluation of the government cash transfer program to determine its effectiveness in improving the health and well-being of orphaned children [21]. Therefore we incorporated sampling households receiving the CT that cared for orphaned and/or separated children to evaluate its effectiveness in UG County. We did not recruit households with ‘only’ vulnerable children as they didn’t fall into the scope of the study’s overall objective and specific aims. For non-CT households, Assistant Chiefs and Village Elders drew up lists of all the households in their villages and sub-Locations caring for orphaned and/or separated children. The lists contained the names of the head of household, their national ID number where available, telephone number where available, the village in which they live, the number of children in the household, and the number of orphaned children in the household. In total from the three sampling arms there were 2,181 households identified; 1,370 from the non cash-transfer arm, and 811 from the CT arm respectively. These lists became the sampling frame for the random selection of SSL, DSL and CT households. The lists were used for stratified random sampling (by Location) of eligible households. Random numbers were generated and assigned using Microsoft Excel Random Number Generator.

Eligible households were required to be caring for orphaned and/or separated children but may also be caring for their own biological children. In order not to ‘single out’ the orphaned child in the household, all children in the household were eligible to participate. In total there were 210 (14.2%) non-orphaned children from households caring for orphans who participated in the study. Households were recruited following extensive community consultations [21,22] and approached individually by Community Health Workers who also validated the eligibility of the household to participate. Consenting, registration, enrolment and all individual study procedures for recruited households took place at the central OSCAR clinic located at Moi Teaching and Referral Hospital (MTRH) in Eldoret.

### Definitions

#### Orphan status

A single orphan was defined as a child whose mother or father was deceased, and a double orphan as one whose parents were both deceased. A separated child was defined as one for whom at least one parent is completely absent from the child’s life [23].

#### Basic material needs

Children’s basic material needs were defined using UNICEF’s definition that each child have at least one blanket, one pair of shoes and two sets of clothing that are not school uniforms [24].

#### Nutritional status

Normal nutritional status was defined as a Z-score of -1 to +2 standard deviations (s.d.) for weight-for-height (for children aged 0-5 years), weight-for-age (for children aged 0-10 years), height-for-age (for children aged 0-18 years), and body mass index-for-age (for children aged 10-18 years). Z-scores greater than or equal to +2 s.d. were defined as over-nutrition. Mild malnutrition was a Z-score of -1 to -2 s.d.. Moderate to severe malnutrition was defined as a Z-score less than or equal to -2 s.d..

### Measures and sources of data

The present analysis utilizes two levels of data: 1) household level data from 300 households that characterized the care environment and 2) individual level data from participants living in households (n = 1481) through a clinical encounter form and for those aged 10 years and above, a psychosocial encounter form.

### Household level data

Household level data were collected through a standardized site assessment to ascertain the characteristics of orphaned and separated children’s care environments. The assessment consisted of 12 sections that covered general characteristics, children in residence, resources, shelter characteristics, guardian characteristics, living and sleeping arrangements, food and meals, material, emotional and psychological needs, policies, family linkages, and household food security. The site assessment was administered in person and in situ to heads of households by trained Community Health Workers. Community Health Workers are residents of the Locations in which they work and have an in-depth knowledge of the households and the cultural context of their communities. As they are representatives of their communities and were introduced by Village Elders, they have developed a rapport and trust with the participants. Due to their trusting relationship and in-depth knowledge of the communities it is likely that respondents are more likely to answer questions honestly. Community Health Workers received extensive and repeated training on administering this questionnaire. The assessment was validated through random household audits.

Household Food Security: was assessed during the site assessment using the household level component of Household Food Insecurity Access Scale (HFIAS) specifically adapted by the USAID Food and Nutrition Technical Assistance (FANTA) project for use in Developing Countries [25]. The HFIAS score is a count measure of the degree of food insecurity in the household over the previous 30 days. A score is calculated for each household by summing the coded frequency of experience for each question. The maximum score for a household is 27, the minimum is 0. The HFIA Prevalence indicator categorizes households into four levels of household food insecurity (access): food secure (score = 0) and mildly food insecure (score = 1-17), moderately food insecure (score = 18-26) and severely food insecure (score > 27).The higher the score, the more food insecurity the household experienced [25].

Sources of material support: was assessed in response to two questions regarding external material support and other sources of income. For both questions respondents could select more than one source of support. External material support included: family, government, religious institutions, other non-governmental organizations, individual sponsors, other, or no external support. Other sources of income included: operating a school, farming, selling vegetables, selling charcoal, shop owner, casual labour, livestock farming, formal employment, begging, commercial sex work, and other.

Other household level variables included: shelter type (temporary, semi-permanent, permanent or other), electricity in the home (yes whole building, yes in some rooms, no), sources of water (river/stream/pond/lake, well/borehole, public standpipe, water piped into the home, purchase bottle water), toilet facilities (pit latrine, indoor flush toilet, none, other), amount of land owned (none, <1/4 acre, ¼-1/2 acre, ½-1 acre, > 1 acre), proportion of household income spent on food (amount of money spent on food / amount of income), household location (rural vs. peri-urban).

#### Individual level data

Socio-demographic and clinical characteristics were assessed and documented through a standardized clinical encounter form and process. The clinical encounter was intended to be an enhanced well-child ‘check-up’ including a complete history and physical review of systems and symptoms. The clinical encounter is administered to children at the OSCAR’s Health and Well Being Project clinic for those living in households in a private space.

School Attendance: was assessed in response to two questions; currently attending school (yes, no, not applicable child not school age) and number of school days missed in the last month (none, 1-2 days, 3-5 days and more than 5 days).

Adequacy of Diet: Adequacy of diet in terms of quantity and quality was assessed by the project nurse using the individual level component of the HFIAS specifically adapted by the USAID FANTA project for use in Developing Countries [25].

Anthropometric measurements: Weight was measured using a digital or infant weighing scale depending on the age of the child and their ability to stand unassisted. It was measured to the nearest 0.1 kg with the child lightly clothed. Height was taken using a heightometer affixed to the wall in the OSCAR Project clinic. The participants stood (without shoes) on a horizontal platform with heels together. They were asked to draw themselves to full height without raising the shoulders, with hands and arms hanging relaxed, and with the feet flat on the ground. The standing body height was measured to the nearest 0.1 cm. For infants, length was measured using an infant heightometer.

Future Outlook: Future outlook was measured by asking participants aged 10 years and above the following in English or Swahili depending on their preference:

“ How do you feel about your future opportunities to be successful and prosper? Would you say:

• Your opportunities are limitless

• You have many opportunities

• Your opportunities are very limited

• You have no opportunities at all”

This question was selected as a measure of subjective well-being [26,27] and because of its increasingly recognized importance as a mediator in mental health, substance use, and self-efficacy [28-30]. This question has been previously used in sub-Saharan Africa through the National Survey of South African Youth [31,32] and specifically in studies of orphaned adolescents also in South Africa [33,34]. For the purposes of binary outcome analysis (logistic regression) we dichotomized responses as opportunities being limitless or many compared to limited or none.

### Statistical analysis

The analysis was restricted to baseline data collected June 2010-April 2013. Parametric and non-parametric descriptive statistics were employed to summarize both categorical and continuous variables. For continuous variables, mean and median together with standard deviation and inter-quartile range were calculated, respectively. Chi-Square test was used to test for associations between categorical /dichotomous variables. Fisher’s exact test was also used if some cells had expected values of less than 5. Missing values for selected covariates are reported. We used inverse probability weighting (IPW) to adjust for missing data on the ‘Future Outlook’ variable. We first used a logistic model for estimating the probability of not having a missing outcome. In addition to covariates included in the outcome model, household type, gender, type of location (rural vs. peri-urban), orphan status, adequacy of diet, educational status, and nutritional indicators were included in the weight model. We used stabilized weights to adjust potential finite-sample bias attributable to having extreme weights. Finally, observations in our outcome model are weighted in inverse proportion to the sampling probability using the inverse of the stabilized weights. Individuals were weighted corresponding to whether they have the observed outcome.

The primary outcomes for this analysis were household socio-economic status, household food security, adequacy of diet, nutritional status, school enrollment, and future outlook. Potential confounders considered a priori and included in the analysis were age and sex of guardian, age and sex of child, and location of the household (peri-urban vs. rural). Post-hoc we additionally tested whether receiving support from family or well-wishers was confounding the effect of the CT. We assessed variables for multicollinearity and found that no pairs of predictors included in our model were highly correlated. There was no inclusion of powers or products of predictor variables in our model.

Nutritional status was based on Z-scores calculated using World Health Organization macros in SAS v. 9.3 for weight-for-height or length (0-5 years), weight-for-age (0-10 years), height- or length-for-age (0-18 years), and body mass index (BMI)-for-age (6-18 years). The reference populations used in these macros are WHO 2007 References for children >5 years and WHO 2006 Child Growth Standards for children < =5 years.

For the food insecurity outcome, we employed Poisson regression model while adjusting for potential confounders such as age, sex of guardian and type of location (peri-urban vs. Rural). We tested for model goodness-of-fit using chi-square test which showed that the model fit the data well. We further tested for over-dispersion using a deviance ratio which was approximately close to one, hence there was no over-dispersion. As such the Poisson model assumptions were adequately met.

Individual-level logistic regression models were also created to examine the association of the CT program with adequacy of diet, future outlook, and moderate-severely low height-for-age, weight-for-age, weight-for-height, and BMI-for-age compared to non-CT households. In these models we adjusted for potential confounding factors: child age, sex, type of location (rural vs. peri-urban), and intra-household clustering using robust standard errors. Analyses were conducted with and without the non-orphaned/non-separated children in the logistic regression models. All analyses were conducted using SAS version 9.3.

## Results

Included in this analysis were data from 300 households (CT: 101, SSL: 89, DSL: 110) and 1481 children and adolescents (CT: 503, SSL: 456, DSL: 522). Overall the population was 50% female, with a median age at enrolment of 13.7 years; 922 (62.3%) were single orphans, 324 (21.9%) were double orphans, and 210 (14.2%) had both parents alive and were living with them.

### Household characteristics

Household-level characteristics including sources of material support and other socioeconomic indicators are summarized in Table 1. As expected given our sampling strategy, the government (through the CT program) supported all CT households; no other households reported government support in any form. Family supported 50% of the CT households, but only 43% of households in SSL, and 26% of households in DSL. Religious institutions provided support to only a small fraction (<5%) of households in each category. While 25% of households in SSL were supported by individual sponsors and well-wishers, only 15% of CT and 10% of DSL households were supported by them. Farming accounted for approximately 50% of the material support of households in each of the three categories, while employment accounted for <3%. Casual labour, selling vegetables, and brewing or selling alcohol were other reported sources of income. Fully 66% of the DSL and 33% of the SSL reported no external support at all, compared to 6% of the CT households.

**Table 1:** Characteristics of households caring for orphaned and separated children participating in the OSCAR Health and Well-Being Study (N = 300)

**Characteristics**	**CT N = 101**	**DSL N = 110**	**SSL N = 89**	**p-value**
	**n (%)**	**n (%)**	**n (%)**	
Sources of material support				
Family	50 (49.5)	28 (25.5)	38 (42.7)	0.010
Government	102 (100)	0 (0.0)	0 (0.0)	<0.001
Religious institutions	4 (4.0)	0 (0.0)	3 (3.4)	xx
Individual sponsors/ well wishers	15 (14.9)	11 (10.0)	22 (24.7)	0.018
Formal employment	2 (2.0)	2 (1.8)	1 (1.1)	0.889
Farming	50 (48.5)	57 (51.8)	52 (58.4)	0.384
Brew/sell alcohol	1 (1.0)	5 (4.6)	4 (4.5)	0.273
Sell vegetables	23 (22.8)	17 (15.5)	18 (20.2)	0.392
Casual labour	17 (16.8)	31 (28.2)	32 (36.0)	0.011
No external support	6 (5.9)	73 (66.4)	29 (32.6)	<0.001
Shelter type				
Temporary	47 (46.5)	46 (41.8)	37 (41.6)	0.785
Semi-permanent	49 (48.5)	58 (52.7)	49 (55.1)	
Permanent	3 (3.0)	6 (5.5)	2 (2.3)	
Missing	2 (2.0)	0 (0.0)	1 (1.1)	
Electricity in home				
In whole building	7 (6.9)	13 (11.8)	2 (2.3)	0.045
In some rooms	2 (2.0)	1 (0.9)	3 (3.4)	
None	89 (88.1)	96 (87.3)	83 (93.3)	
Missing	3 (3.0)	0 (0.0)	1 (1.1)	
Source of water				
Stream/river	12 (11.9)	8 (7.3)	16 (18.0)	0.069
Well/borehole	74 (73.3)	88 (80.0)	58 (65.2)	0.063
Public tap	13 (12.9)	20 (18.2)	17 (19.1)	0.447
Water piped into home	8 (7.9)	9 (8.2)	4 (4.5)	0.542
Toilet facilities				
Pit latrine	92 (91.1)	104 (96.4)	83 (93.3)	0.2125
Indoor flush toilet	0 (0.0)	2 (1.8)	1 (1.1)	
Pit latrine/ Indoor flush toilet	0 (0.0)	2 (1.8)	0 (0.0)	
No toilet facilities	9 (8.9)	4 (3.6)	5 (5.6)	
Amount of land owned				
None	31(30.7)	38 (34.6)	11 (12.4)	0.005
Up to 1 acre	32 (31.7)	33 (30.0)	29 (32.6)	
>1 acre	28 (27.7)	35 (31.8)	38 (42.7)	
Missing	10 (9.9)	4 (3.6)	11 (12.4)	
Average number of meals per day				
Mean (standard deviation)	2.74 (0.44)	2.80 (0.40)	2.67 (0.49)	0.181
Essential material possessions				
Children having ≥ 1 pair of shoes	86 (85.2)	87 (80.6)	76 (85.4)	0.573
Children having ≥ 2 pairs of clothes	77 (76.2)	104 (94.6)	78 (87.6)	0.001
Children having ≥ 1 blanket	22 (22.2)	25 (23.2)	22 (25.0)	0.902
Proportion of household income spent on food				
Expressed as percent	69%	67%	83%	0.183
Household location				
Rural	49 (48.5)	61 (55.5)	52 (58.4)	0.364
Peri-urban	52 (51.5)	49 (44.6)	37 (41.6)	

CT: Cash Transfer, SSL: Same sub-Location (as that receiving the Cash Transfer), DSL: Different sub-Location (from that receiving the Cash Transfer).

The vast majority of housing in each of the three categories was temporary or semi-permanent. The majority of households had no electricity, drew water from a well or borehole, and used pit latrines for toilets. There were 18 households who reported not having even a pit latrine and using the bushes for defecation, including 9 CT and 9 non-CT. Among the CT and DSL households, approximately one-third owned no land, one-third owned up to 1 acre, and 1 third owned more than 1 acre. Among the SSL households, 43% owned more than 1 acre and 33% owned up to 1 acre. There was no difference across the categories in terms of the proportion of income spent on food (represented as percent spent on food: 67%-83%).

Basic material possessions of children were ascertained at the household level for ease of validation (through in situ assessments). There were no significant differences among the household categories in terms of whether all children have at least 1 pair of shoes (81-85%), nor in each child having at least 1 blanket (22-25%). A lower percentage of CT households have all children possessing at least 2 pairs of non-school uniform clothing (76% compared to 88% of SSL and 95% of DSL).

### Household food security

Table 2 details the percentage of households who responded never/rarely vs. sometimes/often to the household food security questions. The mean score for each category of household was 12.7-13.8, and the differences were non-statistically significant. When examined in categories of food secure, mildly food insecure, moderately food insecure, and severely food insecure, similar percentages of households experienced each (Table 3).

**Table 2:** Household Food Insecurity Scores (HFIAS)† among CT, DSL, and SSL households

**In the past 30 days, did:**	**CT**	**DSL**	**SSL**	**P-value**
	**N = 101**	**N = 110**	**N = 89**	
	**n (%)**	**n (%)**	**n (%)**	
You worry that the household (HH) would not have enough food?				
Never/rarely	31(30.7)	21 (19.1)	25 (28.1)	0.128
Sometimes/often	70 (69.3)	89 (80.9)	64 (71.9)	
Was any HH member not able to eat the kinds of foods they preferred because of a lack of resources?				
Never/rarely	29 (29.6)	23 (21.5)	21 (23.6)	0.387
Sometimes/often	69 (70.4)	84 (78.5)	68 (76.4)	
Did any HH member eat just a few kinds of food day after day due to a lack of resources?				
Never/rarely	37 (36.6)	30 (27.3)	23 (25.8)	0.198
Sometimes/often	64 (63.4)	80 (72.7)	66 (74.2)	
Did any HH member eat food that they preferred not to eat because of a lack of resources to obtain other types of food?				
Never/rarely	38 (37.6)	42 (38.2)	39 (43.8)	0.632
Sometimes/often	63 (62.4)	68 (61.8)	50 (56.2)	
Did any HH member eat a smaller meal than you felt the child needed because there was not enough food?				
Never/rarely	40 (39.6)	29 (26.4)	38 (42.7)	0.034
Sometimes/often	61 (60.4)	81 (73.6)	51 (57.3)	
Did any household member eat fewer meals in a day because there was not enough food?				
Never/rarely	40 (39.2)	31 (28.2)	38 (42.7)	0.075
Sometimes/often	61 (60.4)	79 (71.8)	51 (57.3)	
Was there ever no food at all in your household because there were not resources to get more?				
Never/rarely	70 (69.3)	77 (70.0)	68 (76.4)	0.494
Sometimes/often	31 (30.7)	33 (30.0)	21 (23.6)	
Did any HH member go to sleep at night hungry because there was not enough food				
Never/rarely	77 (76.2)	88 (80.0)	77 (86.5)	0.196
Sometimes/often	24 (23.8)	22 (20.0)	12 (13.5)	
Did any HH member go a whole day without eating anything because there was not enough food?				
Never/rarely	78 (77.2)	84 (76.4)	76 (85.4)	0.240
Sometimes/often	23 (22.8)	26 (23.6)	13 (14.6)	
Score	13.01	13.84	12.73	0.340

CT: Cash Transfer, SSL: Same sub-Location (as that receiving the Cash Transfer), DSL: Different sub-Location (from that receiving the Cash Transfer).

†The HFIAS score is a count measure of the degree of food insecurity in the household over the previous 30 days. The maximum score is 27.

**Table 3:** Household Food Insecurity Prevalence by security category among CT, DSL, and SSL Household

		**Household type**		
**Insecurity categories**	**CT**	**DSL**	**SSL**	**p-value**
	**N = 101**	**N = 110**	**N = 89**	
	**n (%)**	**n (%)**	**n (%)**	
Food secure	2 (2.0)	3 (2.7)	1 (1.1)	
Mildly food insecure	75 (74.3)	80 (72.7)	70 (78.7)	
Moderately food insecure	23 (22.8)	27 (24.6)	18 (20.2)	
Severely food insecure	1 (1.0)	0 (0.0)	0 (0.0)	0.893

CT: Cash Transfer, SSL: Same sub-Location (as that receiving the Cash Transfer), DSL: Different sub-Location (from that receiving the Cash Transfer).

### Children’s characteristics

Summarized in detail in Table 4, there were no differences between the three categories of children in their age or gender. CT households had the lowest proportion of children with both parents alive and living with them (i.e. non-orphans), and a higher proportion of single orphans, suggesting a higher burden of orphaned children in the CT households. A similar percentage of children in CT households and those in DSL and SSL households reported having been hospitalized in the past year and currently attending school. There was a significant difference in the number of school days missed in the past month among beneficiaries of the CT program versus the other two categories.

**Table 4:** Individual characteristics of participating children (10-18 years) living in enrolled households (N = 1481)

	**CT**	**DSL**	**SSL**	**P-Value**
	**N = 503**	**N = 522**	**N = 456**	
	**n (%)**	**n (%)**	**n (%)**	
Age in years (median, IQR)	10.3 (5.9-14.1)	10.8 (6.6-14.6)	10.0 (6.7-13.7)	0.328
Missing	0 (0.0)	0 (0.0)	0 (0.0)	
Gender				
Male	231 (45.9)	269 (51.5)	235 (51.5)	0.133
Female	272 (54.1)	253 (48.5)	221 (48.5)	
Missing	0 (0.0)	0 (0.0)	0 (0.0)	
Orphan status				
Single orphan or separated	324 (64.4)	338 (64.8)	260 (57.0)	0.011
Double orphan or separated	113 (22.5)	104 (19.9)	107 (23.5)	
Non-orphaned and living with parents	54 (10.7)	72 (13.8)	84 (18.4)	
Missing	12 (2.4)	8 (1.5)	5 (1.1)	
Education among children aged ≥ 5 years				
Proportion currently in school	370 (93.4)	405 (93.2)	371 (93.2)	0.096
Missing	5 (1.3)	3 (0.7)	2 (0.3)	
Number of days of school missed by child in past 1 month				
None	339 (67.4)	283 (54.2)	251 (55.0)	<0.001
1-2 days	42 (8.4)	76 (14.6)	69 (15.1)	
3-5 days	36 (7.2)	74 (14.2)	64 (14.0)	
>5 days	63 (12.5)	66 (12.6)	65 (14.3)	
Missing	23 (4.6)	23 (4.4)	7 (1.5)	
Quality of diet				
Adequate	465 (92.5)	479 (91.8)	433 (95.0)	0.308
Inadequate	35 (7.0)	37 (7.0)	20 (4.4)	
Missing	3 (0.6)	6 (1.1)	3 (0.7)	
Weight for height (0-5 yrs)				
Normal (Z > -2 to +2)	72 (66.7)	62 (70.5)	46 (76.7)	0.371
Moderately to severely low (Z ≤ -2)	8 (7.4)	10 (11.4)	5 (8.3)	
High (Z > +2)	24 (22.2)	11 (12.5)	6 (10.0)	
Missing	4 (3.7)	5 (5.7)	3 (5.0)	
Height for age (0-18 yrs)				
Normal (Z > -2 to +2)	300 (59.0)	296 (56.3)	218 (47.8)	<0.001
Moderately to severely low (Z ≤ -2)	187 (37.2)	208 (39.5)	227 (49.8)	
High (Z > +2)	16 (3.2)	15 (2.9)	7 (1.5)	
Missing	0 (0.0)	7 (1.3)	4 (0.9)	
Weight for age z scores (0-10 yrs)				
Normal (Z > -2 to +2)	200 (83.3)	181 (75.1)	176 (75.9)	0.114
Moderately to severely low (Z ≤ -2)	30 (12.5)	47 (19.5)	48 (20.7)	
High (Z > +2)	10 (4.2)	13 (5.4)	7 (3.0)	
Missing	0 (0.0)	0 (0.0)	1 (0.4)	
BMI-for-age (6-18 yrs)				
Normal (Z > -2 to +2)	346 (91.8)	377 (90.4)	332 (91.0)	0.235
Moderately to severely low (Z ≤ -2)	19 (5.0)	26 (6.2)	15 (4.1)	
High (Z > +2)	12 (3.2)	8 (1.9)	16 (4.4)	
Missing	0 (0.0)	6 (1.4)	2 (0.6)	
Hospitalized in past 1 year				
Yes	15 (3.0)	11 (2.1)	10 (2.2)	0.603
No	478 (95.0)	505 (97.9)	438 (96.1)	
Missing	10 (2.0)	6 (1.2)	8 (1.8)	
Future outlook (10-18 yrs)				
Limitless or many opportunities	173 (65.8)	144 (51.1)	113 (49.3)	<0.001
Limited or no opportunities	61 (23.2)	80 (28.4)	70 (30.6)	
Missing	29 (11.0)	58 (20.6)	46 (20.1)	

CT: Cash Transfer, SSL: Same sub-Location (as that receiving the Cash Transfer), DSL: Different sub-Location (from that receiving the Cash Transfer).

There were no significant differences in the adequacy of diet or median weight between the three groups, nor in the weight-for-height in the children aged 5 years and under. A higher percentage of children living in CT households than non-CT households had normal height- or length- for their age (0-18 years). There were no differences in the proportion of children with normal weight-for-age or BMI-for-age among the three groups.

Among children aged 10-18 years, although 23%-31% of all children aged 10-18 years in all households reported having no or only limited opportunities in life, a higher proportion (66%) of the CT children (compared to 49% in SSL and 51% in DSL) reported having limitless or many opportunities in life.

#### Adjusted Poisson and logistic regression models

There were no significant differences in the household food security between those receiving the CT versus non-CT households in unadjusted and adjusted analyses (Table 5). Similarly, there were no statistical differences comparing the CT vs. non-CT households in all children having at least 1 pair of shoes or 1 blanket. However, CT households were less likely to have all children possessing at least 2 pairs of non-school-going clothes compared to non-CT households (AOR: 0.32, 95% CI: 0.16-0.63) adjusting for guardian age, sex, and type of location (rural versus peri-urban) (Table 5).

**Table 5:** Poisson Regression analysis and logistic regression analysis comparing household characteristics of cash transfer versus non-cash transfer households on key outcomes

	**All children have >1 blanket**	**All children have >1 pair of shoes**	**All children have >2 pairs of clothes**	**Household food insecurity score**
	**(yes vs. no)**	**(yes vs. no)**		
			**(yes vs. no)**	
Cash transfer vs. non-cash transfer				
Unadjusted odds Ratios (95% confidence intervals)	0.91 (0.51-1.61)	1.20 (0.62-2.32)	0.30 (0.15-0.59)	-0.03 (-0.09, 0.04)
Adjusted* odds ratios	0.89 (0.49-1.60)	0.89 (0.49-1.60)	0.32 (0.16-0.63)	-0.01 (-0.08, 0.05)
(95% confidence intervals)				

*Adjusted for age and sex of guardians and type of location (Peri-urban vs. Rural) for household food insecurity and essential material possessions.

After adjusting for child age, sex and intra-household correlation in the individual level models (Table 6), there were no differences in school attendance (AOR: 0.94, 95% CI: 0.50-1.80) between children in CT households in comparison to non-CT households, but children in CT households were less likely to have missed any days of school in the preceding month (AOR: 0.62, 95% CI: 0.42-0.94). There were no differences in weight-for-height among the under 5’s, or in weight-for-age among children under 10, but those aged <1-18 years in CT-households were less likely to have height stunting for their age compared to the non-CT children (AOR: 0.65, 95% CI: 0.47-0.89). Adolescents at least 10 years of age in CT-households were more likely to have a positive future outlook (AOR: 1.68, 95% CI: 1.10-2.54) than those in non-CT households; this association held when utilizing inverse probability weighting to account for missing data (AOR: 1.72, 95% CI: 1.12-2.65). We conducted these analyses first with only the orphaned and separated participants, and then together with the non-orphan participants in the same households. In general, the results did not change although upon inclusion of the non-orphaned children in the analysis of missing any school in the preceding 30 days, the effect of the CT program became further pronounced, suggesting possible intra-household differences between the orphaned and non-orphaned children.

**Table 6:** Logistic regression analysis (unadjusted and adjusted odds ratios, OR’s, and 95% confidence intervals, CI) comparing individual characteristics of children living in cash transfer households to non-cash transfer households on key outcomes

	**Currently in school (yes vs. no)**	**Number of days of school missed (any vs. none)**	**Adequate diet (vs. inadequate)**	**Weight for Height**	**Height for Age**	**Weight for Age**	**BMI-for-age (moderately or severely low vs. mild/normal/high)**	**Future outlook (limitless/many vs. limited/none)**	**Future Outlook - IPW**
					**(moderately or severely low vs. mild/normal/high)**	**(moderately or severely low vs. mild/normal/high)**			
									**(Inverse Probability Weighting)**
				**(moderately or severely low vs. mild/normal/high)**					
Unadjusted OR (95% CI)	0.81 (0.45-1.46)	0.81 (0.45-1.46)	0.81 (0.45-1.46)	0.81 (0.45-1.46)	0.71 (0.52-0.97)	0.71 (0.52-0.97)	0.71 (0.52-0.97)	0.71 (0.52-0.97)	0.71 (0.52-0.97)
Orphans only	0.94 (0.50-1.80)	0.94 (0.50-1.80)	0.94 (0.50-1.80)	0.94 (0.50-1.80)	0.65 (0.47-0.89)	0.65 (0.47-0.89)	0.65 (0.47-0.89)	0.65 (0.47-0.89)	0.65 (0.47-0.89)
Adjusted* OR									
(95% CI)									
All participants	0.91 (0.49-1.69)	0.91 (0.49-1.69)	0.91 (0.49-1.69)	0.91 (0.49-1.69)	0.70 (0.52-0.96)	0.70 (0.52-0.96)	0.70 (0.52-0.96)	0.70 (0.52-0.96)	0.70 (0.52-0.96)
(orphans & non-orphans)									
Adjusted* OR (95% CI)									

*Adjusted for age and sex of the child and type of location (Peri-urban vs. Rural) plus intra-household clustering effect.

## Discussion

These findings suggest that the Government of Kenya cash transfer program to households caring for orphaned children may be having a measurable positive effect on their school attendance, nutritional status, and future outlook on life and therefore impacting the ability of households to uphold children’s human rights. We did however find significant household food and economic insecurity in all participating households, with 23% of all households reporting moderate food insecurity and only 2% reporting being food secure. This demonstrates large gaps in human rights in relation to access to adequate food despite being in a region of high agricultural productivity.

Over the last decade, Conditional and Unconditional Cash Transfer programs have become one of the most widely adopted anti-poverty initiatives in the developing world [35-40]. They have been successfully used to increase immunization rates, school attendance, and nutritional status among low-income families [41-46], and tested as a mechanism to reduce HIV incidence among high risk adolescent girls [47]. Whether conditionality impacts the efficacy or outcomes of the cash transfer program remains unclear [43]. In the context of children’s human rights, it is important to consider the coverage, sufficiency, and conditionality of cash transfers and the degree to which the scheme is child-oriented [11,12].

Our data add to the growing body of evidence on the association between cash transfers and health and socio-economic outcomes in resource-constrained settings. This paper offers new data on the effect of unconditional cash transfers received by households caring for orphaned children, including the positive associations between receiving the CT and school attendance, nutritional status, future outlook, and some basic material possessions. Conditional cash-transfer programs have proved largely successful in ameliorating school attendance and health outcomes in other resource-constrained settings [14]. It can be hypothesized that school attendance in our study may improve among those receiving the CT due to a decrease in child labour that has occurred in other settings in association with conditional cash-transfers [14], thereby allowing children to regularly attend school. However, the ability of the CT to alleviate poverty as evidenced in our results concerning household food insecurity remains unclear.

The effect of the cash transfers on the future outlook of children and adolescents is another new contribution to the literature. While the mechanism of action is not clear, we hypothesize that children in these households (not just orphaned children) may feel they will have more opportunities later in life because they feel supported by the government and not entirely dependent on their caregivers for support and opportunities. In Brazil, it was found that children benefiting from a conditional cash-transfer program had improved psychosocial health, specifically related to satisfaction in friendships and age-appropriate behavior [48]. The links between psychosocial health outcomes and enrolment in cash-transfer programs needs further exploration using longitudinal and qualitative methods.

There are several strengths to our findings. First, the selection of both cash transfer and non-cash transfer households was random using a well-structured sampling frame for this purpose [21]. This reduces the potential for selection bias and increases the generalizability of our findings. Second, our sampling scheme enabled us to compare households caring for orphans receiving and not receiving the cash transfer subsidy, allowing us to draw direct comparisons. Third, we were able to examine physical and mental health issues as well as schooling and socioeconomic indicators within a relatively robust sample size of hard to reach children and adolescents. Fourth, the use of community health workers to collect household level data may have reduced social desirability bias. Lastly, the use of community-based participatory methods ensured the study was contextually relevant and addressed issues that were important to the participants and policymakers in the region.

There may be limitations to our findings. Many of the outcome measures are self-reported, including household food security, sources of material support, dietary intake (adequacy of diet), and future outlook. They may therefore be subject to various kinds of bias, including reporting bias such as interviewer or social desirability bias. We tried to minimize these through the deployment of Community Health Workers to conduct the household level data collection and by using unannounced household audits. These data may also be subject to random misclassification bias if there were errors in recording or data entry. Third, because these data are cross-sectional, inferences about causality cannot be made. Fourth, the locations and households selected to receive the cash transfers were those considered the neediest, therefore the comparisons between the three categories on socioeconomic indicators should be done with caution. Fifth, there are relatively small numbers in some sub-groups particularly of nutritional status (e.g. height for weight) and these results should also be interpreted with caution. Sixth, the psychosocial indicator ‘future outlook’ should be interpreted with caution as a stand-alone psychosocial indicator and due to the level of comprehension, meaning and interpretation of psychosocial questions may be different between those who are younger (10-14) versus those whom are older (>14) due to their developmental stage and level of educational attainment. Seventh, some households receiving the CT also had external support from individual sponsors and religious organizations that could have altered their socio-economic status and children’s health and well-being outcomes; however, a slightly higher percentage of non-CT households (17%) received this support in comparison to only 15% of CT-households. Eighth, the large time period over which baseline data was collected could alter the ability of the CT program to have an impact on socio-economic and health outcomes indicators measured as those who had a baseline assessment in 2013 had a longer time to benefit from the program. Ninth, we could not take into account how long each family was enrolled in the CT program.

## Conclusion

Children and adolescents in households receiving the CT-OVC appear to have better nutritional status, school attendance, and optimism about the future, compared to those in households not receiving the CT, in spite of some evidence of continued material deprivation. Given the widespread poverty and household food insecurity identified, consideration should urgently be given to further expansion of the program to reach more vulnerable households and strengthen the social safety net for orphans and their caregivers in Kenya.

## Competing interests

The authors declare that they have no competing interests.

## Authors’ contributions

DA, LA, SA, RV, WN, and PB conceived of the research question and study design. DA, LA, SA, RV, WN, AK, and PB were involved with the collection of the data. JK and PB analyzed the data. DA wrote the first draft of the manuscript, LE contributed to manuscript development, and all authors read and approved the final version.

## Pre-publication history

The pre-publication history for this paper can be accessed here:

http://www.biomedcentral.com/1472-698X/14/25/prepub
